# The Protective Effect of Sheng Mai Yin on Diabetic Cardiomyopathy via NLRP3/Caspase-1 Pathway

**DOI:** 10.1155/2022/1234434

**Published:** 2022-12-01

**Authors:** Jing-Ya Li, Chun-Chun Zhao, Jian-Fei Peng, Meng Zhang, Liang Wang, Gang Yin, Peng Zhou

**Affiliations:** ^1^School of Integrated Chinese and Western Medicine, Anhui University of Chinese Medicine, Hefei 230012, China; ^2^The Second Affiliated Hospital of Anhui University of Chinese Medicine, Hefei, China

## Abstract

Sheng Mai Yin (SMY) has therapeutic effects on myocardial infarction (MI), heart failure (HF), diabetic cardiomyopathy (DCM), and myocarditis. To study whether SMY can relieve pyroptosis and play a protective role in diabetic cardiomyopathy, a molecular docking technique was used to predict the possible mechanism of SMY against DCM. Then, a DCM rat model was induced by intraperitoneal injection of streptozotocin (STZ), divided into 5 groups: the DM group (model), SMY-L group (2.7 mL/kg SMY), SMY-M group (5.4 mL/kg SMY), SMY-H group (10.8 mL/kg SMY), and Met group (120 mg/kg metformin). Rats in the CTL group (control) and DM group were given normal saline. After 8 weeks, the levels of blood glucose, lipids, and myocardial enzymes were detected according to the kit instructions. Cardiac function was detected by echocardiography. HE and Masson were used to observing the pathological changes, collagen deposition, and collagen volume fraction (CVF). The apoptosis rate of cardiomyocytes was determined by Tunel. The IL-1*β* level was determined by ELISA and RT-PCR. The expressions of NLRP3, caspase-1, and GSDMD were measured using RT-PCR and Western blotting. The docking results suggested that SMY may act on NLRP3 and its downstream signal pathway. The *in vivo* results showed that SMY could reduce blood glucose and lipid levels, improve heart function, improve histopathological changes and myocardial enzymes, and alleviate cardiomyocyte apoptosis and myocardial fibrosis. SMY inhibited the mRNA and protein expressions of NLRP3, ASC, Caspase-1, and GSDMD and IL-1*β* production. SMY can reduce DCM by regulating the NLRP3/caspase-1 signaling pathway, providing a new research direction for the treatment of DCM.

## 1. Introduction

Diabetic cardiomyopathy (DCM) is a specific cardiac manifestation of diabetes patients and is the main cause of morbidity and mortality of diabetes patients around the world. In China, 33.9% of patients with type 2 diabetes have cardiovascular disease [1]. The pathogenesis of DCM involves cardiac inflammation and changes in metabolic characteristics, characterized by early left ventricular hypertrophy and diastolic dysfunction, manifested in myocardial cell hypertrophy, apoptosis, and myocardial interstitial fibrosis. With the progression of the disease, DCM gradually evolved into systolic dysfunction with reduced ejection fraction and eventually developed into heart failure [2, 3]. DCM is characterized by a variety of pathophysiological variables, including oxidative stress, apoptosis, cardiac fibrosis, impaired angiogenesis, and altered glycolysis metabolism, although its pathogenesis is unknown. Presently, there is no particular medicine available for the treatment of DCM, and the majority of patients suffer from heart failure. Identifying possible treatment targets is crucial for reducing the morbidity and mortality associated with DCM.

Numerous studies have demonstrated that the nucleotide-binding domain leucine-rich repeat (NLR) and pyrin domain-containing receptor 3 (NLRP3) inflammasome play a significant role in the pathophysiology and research advancement of DCM and is a new pharmacological target for treating DCM and related complications [4, 5]. In individuals with DCM, activation of the NLRP3 inflammasome has been found to generate or exacerbate cardiac inflammation, myocardial cell necrosis or apoptosis, myocardial fibrosis, and cardiac failure [6, 7].

Sheng Mai Yin (SMY) is composed of ginseng, radix ophiopogonis, and *Schisandra chinensis*. Some studies have shown that the Sheng Mai compound preparation composed of three Chinese herbs has therapeutic effects on heart diseases such as myocardial ischemia-reperfusion, coronary heart disease, and heart failure [8–10]. By activating AMPK*α* and decreasing oxidative stress injury mediated by NADPH oxidase, Sheng Mai San (SMS) has been reported to protect the myocardium against diabetes [11]. Through stimulation of the Nrf2/Keap1 signaling pathway, Sheng Mai injection (SMI) inhibits apoptosis, decreases CK, LDH, and MDA levels, and increases SOD activity, therefore reducing DOX-induced cardiotoxicity [12]. However, the mechanism of action of SMY on DCM is yet unknown, and it has to be determined whether SMY may play a protective function in DCM by acting on the NLRP3/Caspase-1 pathway.

## 2. Materials and Methods

### 2.1. Molecular Docking

CB-Dock was used to predict the binding affinity between the main ingredients of SMY and NLRP3. The major components of SMY were 11 chemical components found in rat serum: ginsenoside Rg1, ginsenoside Re, ginsenoside Rf, ginsenoside Rg2, ginsenoside Rb1, ginsenoside Rd, ginsenoside Rc, ophiopogonin D, schisandrin, schisandrol B, and schisandrin B [13]. The structures were downloaded from the PubChem website and then the hydrogens and charge were added. The PDB format of NLRP3 (ID: 6npy) [14] was downloaded from RCSB (https://www.rcsb.org/), waters and het groups were deleted, and hydrogens were added, and then the docking procedure was submitted [15].

### 2.2. Animals

Male Sprague-Dawley (SD) rats were purchased from Pizhou Oriental Breeding Co., Ltd., SCXK (Su) 2017-0003. All procedures have been approved by the Center for Scientific Research of Anhui University of Chinese Medicine (AHUCM-rats-2021076).

### 2.3. Chemicals and Materials

SMY (20260504) was purchased from Beijing Tongrentang Science and Technology Development Co., Ltd. Metformin hydrochloride (67190501) was purchased from Shanghai Xinyi Tianping Pharmaceutical Co., Ltd. Streptozotocin (STZ, CAT.No. 2196GR001) was purchased from BIOFROXX, Germany. Cardiac troponin I (CTNI, Cat.#RX301624R), glycosylated hemoglobin (GHB, Cat.#RX302312R), atrial natriuretic peptide (ANP, Cat.#RX302231R), B-type natriuretic peptide (BNP, Cat.#RX302959R), and interleukin-1*β* (IL-1*β*, Cat.#RX302869R) were purchased from Quanzhou RUIXIN Biotechnology Co., Ltd. Total cholesterol (TC, A111-1-1), creatine kinase (CK, A032-1-1), and low-density lipoprotein cholesterol (LDL-C, A113-1-1) were purchased from Nanjing Jiancheng Institute of Biological Engineering. Triglyceride (TG, C061-A) and glucose assay kits (C050-g) were purchased from Changchun Huili Biotechnology Co., Ltd. High-density lipoprotein cholesterol (HDL-C, A0-10137) was purchased from Zhejiang Dongou Diagnostic Products Co., Ltd. Anti-NLRP3 (ab263899) and anti-GSDMD (ab219800) were purchased from Abcam (Cambridge, MA, United States). Anticleaved-Caspase 1 (AF4005), antitubulin (AF7011), and anti-ASC (DF6304) were purchased from Affinity Biosciences (Cincinnati, OH, USA). Anti-GAPDH (380626) was purchased from Chengdu Zen Biotechnology Co., Ltd. The reverse transcription kit (BL699A) was purchased from BioSharp, China. SYBR Green (G3326-05) was purchased from Servicebio, China.

### 2.4. Establishment of the Rat Model

After a week of adaptive feeding, 10 male SD rats were chosen at random for the CTL group and fed a standard diet. Other rats were fed a high-calorie diet (composition: lard 5%, sugar 5%, yolk powder 5%, and cholesterol 1%). Each group of rats was fed routinely for six weeks. Rats in the control group were treated intraperitoneally with citric acid-sodium citrate buffer (pH 4.2) after 6 weeks, whereas rats in the other groups were injected intraperitoneally with 1% STZ at 35 mg/kg. After 3 days, blood was taken by needling at the tail tips of rats in each group, and the diabetic model was successfully prepared when the blood glucose was ≥16.7 mmol/L [16].

### 2.5. Drug Treatment

Successfully modeled SD rats were randomly assigned to five groups: DM (model), SMY-L (2.7 mL/kg SMY), SMY-M (5.4 mL/kg SMY), SML-H (10.8 mL/kg SMY), or Met (120 mg/kg metformin). Both the CTL group and the model group received the same amount of normal saline. All groups received the same gavage intervention for eight weeks.

### 2.6. Detection of Blood Glucose and Lipid Levels

After fasting for 12 h, rats in each group were anesthetized with pentobarbital sodium, and blood was taken from the abdominal aorta to the vein collection. After 30 min, the supernatant was centrifuged at 1500 r/min for 15 min and stored at −80°C. Blood glucose, GHB, TG, TC, LDL-C, and HDL-C values were measured according to the kit's instructions.

### 2.7. Detection of Cardiac Function by Echocardiography

All rats were anesthetized with isoflurane and administered 2D echocardiography for calculating ejection fraction (EF) and fractional shortening (FS) [16].

### 2.8. Detection of Pathological Staining

The myocardial tissue was fixed in 4% paraformaldehyde at room temperature, dehydrated, embedded in paraffin, and sliced into 5 *μ*m sections. Different sections were stained with HE and Masson and observed under a microscope, which was used to observe the pathological changes, collagen deposition in the myocardial interstitium, and collagen volume fraction (CVF).

### 2.9. Detection of Myocardial Enzymes

The contents of CK, ANP, and BNP were detected according to the kit's instructions.

### 2.10. Detection of Apoptosis Rate

In each slice, five visual fields were randomly selected for microscopic examination, and the total number of cardiomyocytes and the apoptotic number of cardiomyocytes were counted in each field. Apoptosis rate = apoptotic cardiomyocytes number/all cardiomyocytes number × 100%.

### 2.11. Detection of IL-1*β* Levels

IL-1*β* levels in rat myocardial tissue were detected by a commercial test kit. The optical density (OD) was selected at 450 nm and read by a microplate reader (BioTek, USA).

### 2.12. Detection of mRNA Expression by RT-PCR

Myocardial RNA was extracted from the left ventricular myocardium using the Trizol reagent. Using a reverse transcription kit, an equal quantity of RNA from each sample was reversely transcribed into cDNA. For PCR amplification of the same number of reverse transcription products, SYBR Green was used. RT-PCR was conducted with the LightCycler® 96 PCR apparatus (Roche, Switzerland). The results were analyzed using 2^−ΔΔCq^ method to evaluate the mRNA levels of NLRP3, ASC, Caspase-1, and GSDMD. The primers used in the research are shown in Table 1.

### 2.13. Detection of Protein Expression by Western Blotting

The heart tissue was lysed in lysate, and total protein was extracted. On a 10% to 15% polyacrylamide gel, the lysates were separated and transferred to an NC membrane. After blocking the NC membrane with 5% skim milk powder, the following antibodies were incubated at 4°C overnight: anti-NLRP3 (1 : 1000), anticleaved caspase-1 (1 : 1000), anti-ASC (1 : 1000), anti-GSDMD (1 : 1000), anti-GAPDH (1 : 5000), and anti-tubulin (1 : 5000). The primary antibody was incubated at 4°C for an overnight before being incubated at room temperature with the secondary antibody for 2 h. The density of protein bands was detected using the ECL chemical substrate luminescence kit, and the protein bands were imaged in Tanon5200 imaging system (Tanon, China).

### 2.14. Statistical Analysis

The data were given as mean ± standard deviation (SD), and statistical analysis was performed using SPSS 23.0. A one-way ANOVA was utilized to determine the significance between the groups. *P* < 0.05 was regarded as statistically significant.

## 3. Results

### 3.1. Docking Results

**Figure 1:**
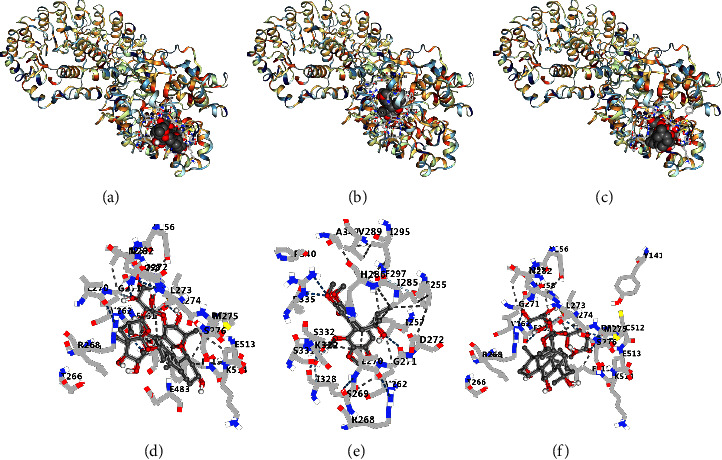
The docking pictures of the main chemical constituents of SMY with NLRP3. (a) Ophiopogonin D-NLRP3. (b) Schisandrol B-NLRP3. (c) Ginsenoside Rf-NLRP3. (d) Ophiopogonin D-amino acid residues. (e) Schisandrol B-amino acid residues. (f) Ginsenoside Rf-amino acid residues.

The primary components of SMY exhibited binding affinity with NLRP3. The superior binding affinities of Ophiopogonin D, Schisandrol B, and Ginsenoside Rf to NLRP3 may provide active monomer compounds for future investigation (Table 2 and Figure 1). The docking data revealed that SMY may operate on NLRP3 and its downstream signaling pathway, which was confirmed by *in vivo* tests.

### 3.2. SMY Reduced Blood Glucose and Lipid Levels in Model Rats

**Figure 2:**
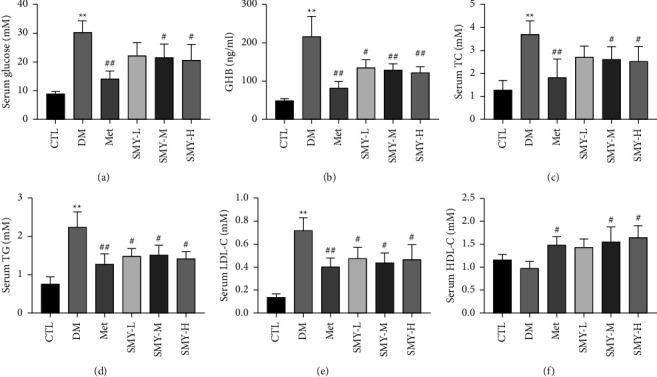
SMY reduced blood glucose and lipid levels in model rats. (a) Blood glucose. (b) GHB. (c) TC. (d) TG. (e) LDL-C. (f) HDL-C. The values were expressed as the mean ± SD (*n* = 10), ^*∗∗*^*P* < 0.01 vs. CTL group, ^#^*P* < 0.05, ^##^*P* < 0.01 vs. DM group.

The abnormal blood glucose and blood lipid levels suggested that the DCM model had been developed successfully [17]. After 8 weeks, the levels of blood glucose, GHB, TG, TC, and LDL-C were substantially higher in the DM group than in the CTL group (*P* < 0.01), although the level of HDL-C did not change significantly. After therapy with SMY-M and SMY-H, blood glucose, GHB, TG, TC, and LDL-C levels were reduced (*P* < 0.05, *P* < 0.01) (Figure 2). Rat models demonstrated that SMY may successfully lower blood glucose and blood lipid levels.

### 3.3. SMY Improved the Heart Function in Model Rats

**Figure 3:**
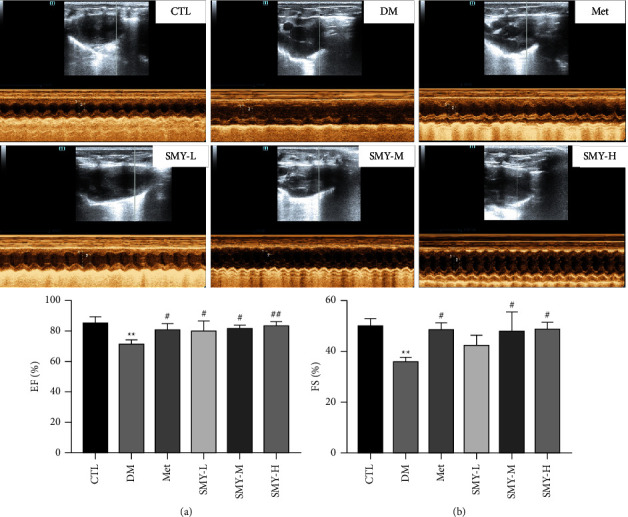
SMY improved the heart function of DCM rats. (a) EF (%). (b) FS (%). The values were expressed as the mean ± SD (*n* = 10), ^*∗∗*^*P* < 0.01 vs. CTL group, ^#^*P* < 0.05, ^##^*P* < 0.01 vs. DM group.

Echocardiography was performed to determine the heart function of each group of rats, with EF and FS serving as the primary indices. The EF and FS of the DM group were considerably lower than those of the CTL group (*P* < 0.01), while SMY and Met reversed the FS and LVEF (*P* < 0.05, *P* < 0.01) (Figure 3). The results suggested that SMY has a certain ameliorative effect on diabetic cardiac function injury.

### 3.4. SMY Improved the Histopathological Changes and Myocardial Enzymes in Model Rats

**Figure 4:**
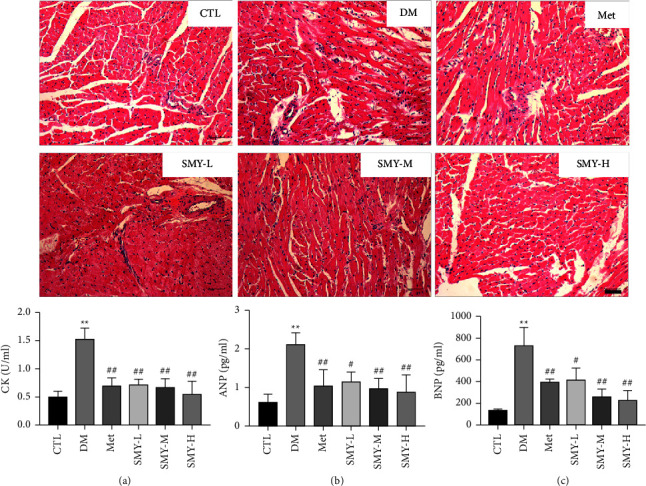
SMY improved the histopathological changes and myocardial enzymes in model rats. (a) CK. (b) ANP. (c) BNP. The values were expressed as the mean ± SD (*n* = 10), ^*∗∗*^*P* < 0.01 vs. CTL group, ^#^*P* < 0.05, ^##^*P* < 0.01 vs. DM group.

The findings of the HE staining demonstrated that the CTL group possessed normal morphology, a full myocardial structure, and an organized arrangement of muscle fibers. The DM group had myocardial fracture, myocardial fiber organization abnormality, and inflammatory cell infiltration. The myocardial fibers of the Met group and the SMY group were arranged in a relatively orderly manner with a small amount of inflammatory infiltration. Creatine kinase (CK) is mainly used in the diagnosis of myocardial infarction, which is widely in skeletal muscle, cardiac muscle, and brain tissue. Serum atrial natriuretic peptide (ANP) and B-type natriuretic peptide (BNP) activities are used to diagnose and monitor the course and efficacy of heart failure [18]. CK, ANP, and BNP levels were considerably greater in the DM group than in the CTL group (*P* < 0.01); SMY and Met lowered CK, ANP, and BNP levels (*P* < 0.05, *P* < 0.01) (Figure 4). The results showed that SMY could significantly reduce the changes in cardiac pathology and myocardial enzymes in model rats.

### 3.5. SMY Alleviates Cardiomyocyte Apoptosis and Myocardial Fibrosis in Model Rats

**Figure 5:**
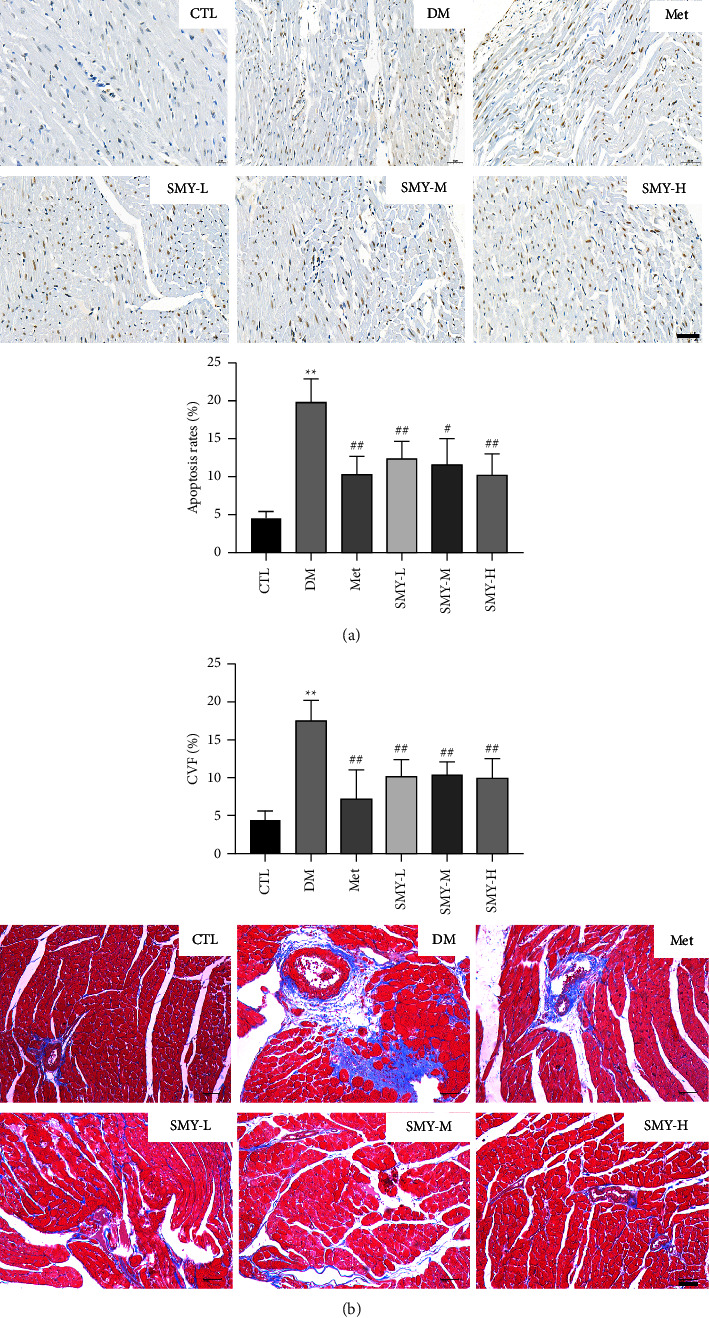
SMY alleviated cardiomyocyte apoptosis and myocardial fibrosis in model rats. (a) Tunel. (b) Masson. The values were expressed as the mean ± SD (*n* = 10), ^*∗∗*^*P* < 0.01 vs. CTL group, ^#^*P* < 0.05, ^##^*P* < 0.01 vs. DM group.

The DM group's nuclei were discovered to be pale brown, and their apoptosis rate was significantly greater than that of the CTL group (*P* < 0.01). Compared to the DM group, SMY and Met treatment significantly decreased the amount of pale brown nuclei and the apoptosis rate (*P* < 0.05, *P* < 0.01) (Figure 5(a)). Masson staining revealed that the muscle fibers of the myocardium were red and the collagen fibers were blue. In the CTL group, the myocardial fibers were organized and only a modest number of collagen fibers were formed, whereas the DM group had abundant collagen fiber depositions. Pretreatment with SMY and Met resulted in a significant decrease in the collagen content of cardiac tissue. Collagen volume fraction (CVF) can be utilized to examine morphological alterations of the left ventricular myocardium. CVF levels were considerably greater in the DM group than in the CTL group (*P* < 0.01), but SMY and Met lowered CVF levels (*P* < 0.01) (Figure 5(b)).

### 3.6. SMY Decreased IL-1*β* Level in Rat Myocardial Tissue

**Figure 6:**
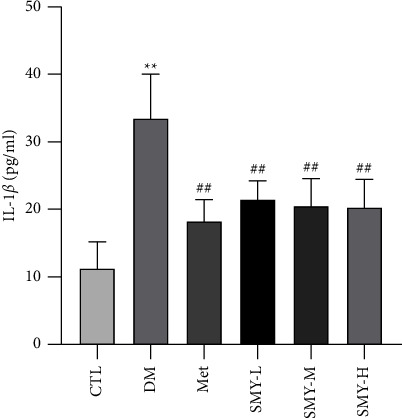
SMY decreased IL-1*β* Level in rat myocardial tissue. The values were expressed as the mean ± SD (*n* = 10), ^*∗∗*^*P* < 0.01 vs. CTL group, ^##^*P* < 0.01 vs. DM group.

As shown in Figure 6, IL-1*β* level in rat myocardial tissue increased significantly in the DM group compared with the CTL group (*P* < 0.01). IL-1*β* level was decreased significantly in the Met and SMY groups (*P* < 0.05).

SMY inhibited the mRNA expressions of NLRP3, ASC, caspase-1, GSDMD, and IL-1*β*.

**Figure 7:**
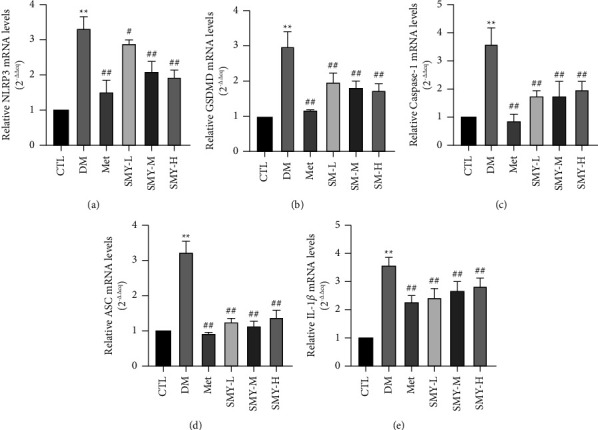
SMY inhibited the mRNA expressions of NLRP3, ASC, caspase-1, GSDMD, and IL-1*β*. (a) NLRP3. (b) GSDMD. (c) Caspase-1. (d) ASC. (e) IL-1*β*. The values were expressed as the mean ± SD (*n* = 3), ^*∗∗*^*P* < 0.01 vs. CTL group, ^#^*P* < 0.05, ^##^*P* < 0.01 vs. the DM group.

Compared to the CTL group, the mRNA expressions of NLRP3, ASC, caspase-1, GSDMD, and IL-1*β* increased considerably in the DM group (*P* < 0.01). In contrast to the DM group, SMY and Met were able to reverse the mRNA overexpressions (*P* < 0.05, *P* < 0.01) (Figure 7).

### 3.7. SMY Inhibited Protein Expressions of NLRP3, ASC, Caspase-1, and GSDMD

**Figure 8:**
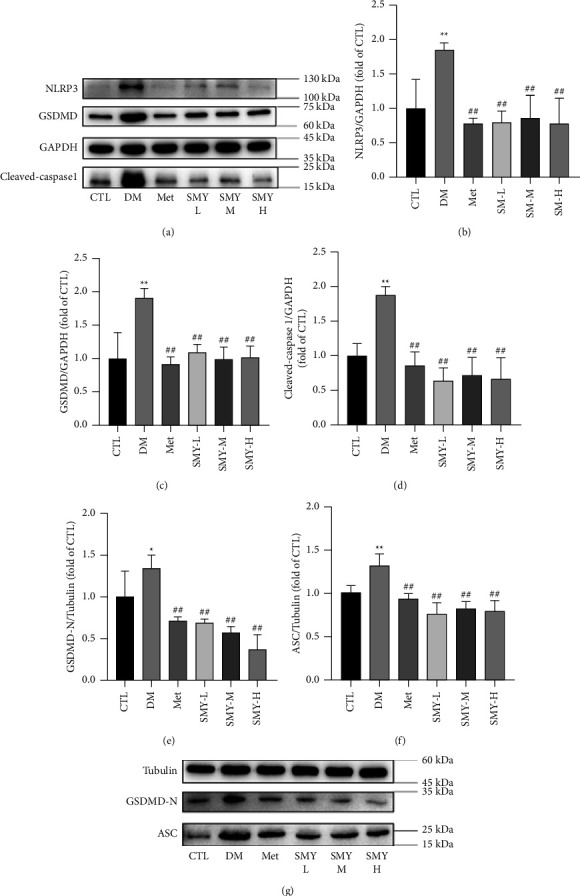
SMY inhibited the protein expressions of NLRP3, ASC, caspase-1, GSDMD, and GSDMD-N. (a) Protein bands of NLRP3, GSDMD, and the cleaved caspase-1. (b) NLRP3. (c) GSDMD. (d) Cleaved caspase-1. (e) GSDMD-N. (f) ASC. (g) Protein bands of GSDMD-N and ASC. The values were expressed as the mean ± SD (*n* = 3), ^*∗*^*P* < 0.05, ^*∗∗*^*P* < 0.01 vs. CTL group, ^##^*P* < 0.01 vs. the DM group.

Protein expressions of NLRP3, ASC, caspase-1, GSDMD, and GSDMD-N in the DM group increased considerably compared to the CTL group (*P* < 0.05, *P* < 0.01). SMY and Met decreased the protein expressions of NLRP3, ASC, caspase-1, GSDMD, and GSDMD-N relative to the DM group (*P* < 0.01). These results suggest that SMY may play a protective role in the myocardium by acting on the NLRP3/caspase-1/GSDMD signaling pathway (Figure 8).

## 4. Discussion

DCM is a diabetes-related pathophysiological disorder characterized by structural, functional, and metabolic abnormalities in the heart that can lead to HF in the absence of coronary artery disease, hypertension, and valvular heart disease [19]. Hyperglycemia, insulin resistance, hyperinsulinemia, and increased free fatty acid metabolism cause oxidative stress, inflammation, formation of advanced glycation end products, abnormal calcium homeostasis, and apoptosis, resulting in myocardial cell dysfunction, injury, and death and consequently cardiac dysfunction [20]. The underlying molecular mechanism of DCM is not yet fully understood. During the development of DCM, excessive hyperglycemia can cause an increase in reactive oxygen species, which activate NF-*κ*B and subsequently trigger the activation of NLRP3, driving cellular inflammation and apoptosis [21]. NLRP3 is an immune-related inflammatory molecule, and studies have established its strong association with the development of DCM [22]. By activating p65, ROS stimulates the activation of NLRP3. Activated NLRP3 interacts with the adaptor protein apoptosis-associated speck-like protein (ASC) and procaspase-1 to create the NLRP3 inflammasome and innate immune system protein complexes [23]. Then, caspase-1 is the effector protein of the NLRP3 inflammasome, cleaved by the precursor molecule procaspase-1 [24]. Caspase-1 may result in the cleavage of pyroptosis executioner gasdermin D (GSDMD) along the canonical pathway. The GSDMD-N possesses membrane pore-forming activity by attaching to phosphoinositides in the plasma membrane, resulting in pyroptosis [25].

As we all know, Met is the first choice for a hypoglycemic drug in the treatment of type 2 diabetes. The UK Prospective Diabetes Study (UKPDS) confirmed that Met can reduce the progress of cardiovascular disease (UK Prospective Diabetes Study [26]. Previous studies have shown that Met could improve cardiac function damage [27] and play a cardiac protective role in diabetes [28, 29]. In this study, we found that SMY had the same effect as Met in improving cardiac function damage, which led us to believe that SMY can protect the heart from diabetes. SMY, a traditional Chinese medicine, is commonly used in the clinic to treat cardiac insufficiency, coronary heart disease, and heart failure [8, 9, 30]. SMY could restore the cardiac function of CHF rats, reduce serum biochemical indexes, reduce cardiac tissue damage, and reduce the expression levels of ALOX15 and CYP1A2, which may be related to the linoleic acid metabolic pathway [10]. SMY could reduce aortic plaque area and MMP9 expression in animal models of myocardial ischemia and atherosclerosis (AS) in response to DEP exposure. In addition, SMY also could improve left ventricular structure, morphology, function, blood flow, infarct area, myocardial damage, and ROS accumulation to varying degrees in ApoE^−/−^ mice, which had a potential protective effect in DEP-aggravated AS with myocardial ischemia [31]. Hence, SMY demonstrates potential cardiac protective actions, including antilipid peroxidation and antiinflammatory characteristics, scavenging oxygen free radicals, antiischemia and hypoxia and cardiomyocyte protection, and regulation of linoleic acid metabolism [10, 31, 32]. Eleven components of SMY in rat serum were determined by LC-MS/MS, which were ginsenoside Rg1, ginsenoside Re, ginsenoside Rf, ginsenoside Rg2, ginsenoside Rb1, ginsenoside Rd, ginsenoside Rc, ophiopogonin D, schisandrin, schisandrol B, and schizandrin B [13]. Several compounds have been discovered to have potent inhibitory effects on the NLRP3 signaling pathway, providing an experimental foundation for the investigation of SMY. Ginsenoside Rg1 improved cardiac function and suppressed lipopolysaccharide (LPS)-induced apoptosis and inflammation in mice. These effects were due to the regulation of the increased expression of toll-like receptor 4 (TLR4), NF-*κ*B, and NLRP3 [33]. Ginsenoside Re reduced the elevated NLRP3, ASC, and caspase-1 protein expression in the hippocampus of mice with chronic restraint stress (CRS) [34]. Ginsenoside Rb1 inhibited the production of TNF-*α*, IL-18, and IL-1*β* in the hippocampus, reduced the protein expression of NLRP3, and stimulated the protein expressions of Nrf2 and HO-1 [35]. Ginsenoside Rd reduced significantly the activation of the NLRP3 inflammasome, which is reliant on the mitochondrial translocation of p62 and mitophagy [36]. In addition, we predicted the binding affinities of 11 chemicals in SMY with NLRP3 using molecular docking and discovered that they all exhibited excellent binding ability. The superior binding affinities of ophiopogonin D, schisandrol B, and ginsenoside Rf to NLRP3 may provide active monomer compounds for future investigation.

**Figure 9:**
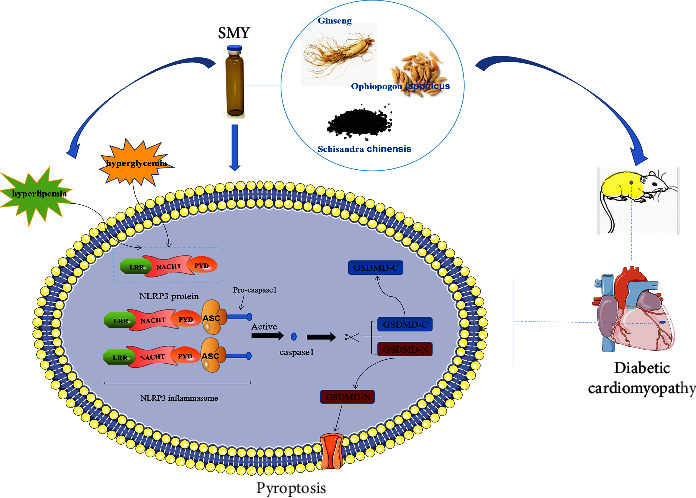
The protective effect of SMY on diabetic cardiomyopathy is based on the NLRP3/caspase-1 pathway.

Molecular docking indicated in this work that SMY may interact with NLRP3 and its downstream signal pathway. *In vivo* studies demonstrated that SMY can alleviate the symptoms of DCM by lowering blood glucose and cholesterol levels, enhancing heart function, histological alterations, and myocardial enzyme and decreasing cardiomyocyte apoptosis and myocardial fibrosis. These pharmacodynamic findings imply that SMY might greatly protect the myocardium against DCM. SMY might also suppress the mRNA and protein expressions of targets in the NLRP3/caspase-1 signaling pathway in DCM (Figure 9). The results revealed that SMY can prevent and treat DCM and that its protective impact is connected to its NLRP3/caspase-1 regulatory signaling pathway.

## 5. Conclusion

The effects of the SMY on the DCM *in vivo* have been verified. SMY might greatly reduce the incidence and progression of DCM in terms of cardiac function, myocardial enzymes, histology, apoptosis, and the signaling pathway. SMY's effect on DCM is mediated through the NLRP3/caspase-1 signaling pathway. However, whether its mechanism is directly related to the NLRP3/caspase-1 signaling pathway needs further experimental verification. The next research will focus on *in vitro* cellular mechanisms, such as adding a specific inhibitor of NLRP3 or using NLRP3 gene silencing in the experiment, in an attempt to better clarify the mechanism of SMY in the prevention and treatment of DCM and provide a new research direction for the treatment of DCM.

## Figures and Tables

**Table 1:** Primers used in RT-qPCR.

Primers		Sequence (5' ⟶ 3′)
NLRP3	Forward	5′-GAGCTGGACCTCAGTGACAATGC-3′
	Reverse	5′-AGAACCAATGCGAGATCCTGACAAC-3′

Caspase-1	Forward	5′-GCACAAGACTTCTGACAGTACCTTCC-3′
	Reverse	5′-GCTTGGGCACTTCAATGTGTTCATC-3′

GSDMD	Forward	5′-CAGCAGGCAGCATCCTTGAGTG-3′
	Reverse	5′-CCTCCAGAGCCTTAGTAGCCAGTAG-3′

ASC	Forward	5′-ATGGTTTGCTGGATGCTCTGTATGG-3′
	Reverse	5′-AAGGAACAAGTTCTTGCAGGTCAGG-3′

IL-1*β*	Forward	5′-AATCTCACAGCAGCATCTCGACAAG-3′
	Reverse	5′-TCCACGGGCAAGACATAGGTAGC-3′

*β*-actin	Forward	5′-CCCATCTATGAGGGTTACGC-3′
	Reverse	5′-TTTAATGTCACGCACGATTTC-3′

**Table 2:** Docking of the main chemical constituents of SMY with NLRP3.

Chemicals	Vina score	Cavity score	Center (*x*, *y*, *z*)	Size (*x*, *y*, *z*)
Ophiopogonin D	−9.0	12730	88, 94, 81	35, 34, 35
Schisandrol B	−8.4	12730	88, 94, 81	35, 34, 35
Ginsenoside Rf	−6.8	12730	88, 94, 81	35, 34, 35
Ginsenoside Re	−6.7	12730	88, 94, 81	35, 34, 35
Ginsenoside Rg2	−6.7	12730	88, 94, 81	35, 34, 35
Schizandrin B	−6.6	12730	88, 94, 81	35, 34, 35
Ginsenoside Rg1	−6.3	12730	88, 94, 81	35, 34, 35
Ginsenoside Rd	−6.2	12730	88, 94, 81	35, 34, 35
Schisandrin	−6.1	12730	88, 94, 81	35, 34, 35
Ginsenoside Rb1	−5.8	12730	88, 94, 81	35, 34, 35
Ginsenoside Rc	−4.9	12730	88, 94, 81	35, 34, 35

## Data Availability

The data used to support the findings of this study are available from the corresponding author upon request.
